# Radioprotective Effect of Aminothiol PrC-210 on Irradiated Inner Ear of Guinea Pig

**DOI:** 10.1371/journal.pone.0143606

**Published:** 2015-11-23

**Authors:** Arnaud P. J. Giese, Jess G. Guarnaschelli, Jonette A. Ward, Daniel I. Choo, Saima Riazuddin, Zubair M. Ahmed

**Affiliations:** 1 Department of Otorhinolaryngology Head & Neck Surgery, School of Medicine, University of Maryland, Baltimore, Maryland, United States of America; 2 Department of Radiation Oncology, University of Cincinnati, Ohio, United States of America; 3 Department of Radiation Oncology, TriHealth Cancer Institute, Cincinnati, Ohio, United States of America; 4 Division of Pediatric Otolaryngology Head & Neck Surgery, Cincinnati Children’s Hospital Medical Center, Cincinnati, Ohio, United States of America; Instituto Nacional do Câncer, BRAZIL

## Abstract

Radiotherapy of individuals suffering with head & neck or brain tumors subserve the risk of sensorineural hearing loss. Here, we evaluated the protective effect of Aminothiol PrC-210 (3-(methyl-amino)-2-((methylamino)methyl)propane-1-thiol) on the irradiated inner ear of guinea pigs. An intra-peritoneal or intra-tympanic dose of PrC-210 was administered prior to receiving a dose of gamma radiation (3000 cGy) to each ear. Auditory Brainstem Responses (ABRs) were recorded one week and two weeks after the radiation and compared with the sham animal group. ABR thresholds of guinea pigs that received an intra-peritoneal dose of PrC-210 were significantly better compared to the non-treated, control animals at one week post-radiation. Morphologic analysis of the inner ear revealed significant inflammation and degeneration of the spiral ganglion in the irradiated animals not treated with PrC-210. In contrast, when treated with PrC-210 the radiation effect and injury to the spiral ganglion was significantly alleviated. PrC-210 had no apparent cytotoxic effect *in vivo* and did not affect the morphology or count of cochlear hair cells. These findings suggest that aminothiol PrC-210 attenuated radiation-induced cochlea damage for at least one week and protected hearing.

## Introduction

Radiation is a treatment modality used for tumors of the head, neck and central nervous system. One of the clinical challenges of radiation to these sites is that the inner ear (cochlea and vestibular organs), temporal bone, and brain are within the radiation field, which may result in sensorineural hearing loss and balance disorders [1, 2]. Generally, radiation induced sensorineural hearing loss is a result of degeneration of the outer and inner hair cells within the organ of Corti and spiral ganglion neurons [3]. Many attempts have been made to prevent, minimize, or reverse the effects of radiation on the inner ear. The greatest advances have been achieved by improved radiation techniques that have reduced radiation dose to the inner ear [4].

Patients treated with therapeutic radiation for brain tumors, acoustic neuromas, or nasopharyngeal carcinomas may develop radiation-induced hearing loss due to damage of the inner ear [5, 6]. *In vitro* studies have revealed that radiation induces the production of reactive oxygen species (ROS). These ROS create an oxidative stress that activates the apoptotic pathway, which ultimately leads to the degeneration of sensory hair cells, cells in the stria vascularis, and the spiral ganglion neurons [7]. For decades, efforts have been made to determine effective radioprotective agents that may protect the inner ear in animal models or *in vitro* hair cell lines [7]. These radioprotective agents include Epicatechin [8], Amifostine [9], L-N-acetylcysteine [10], L-carnitine [11] and piracetam [12]. Some of these compounds may interfere with cancer treatment, as they were not tested on animal tumor models. Amifostine and palifermin are radioprotective compounds that scavenge free radicals and inhibit apoptosis and are the only two compounds approved by the US FDA in radiation therapy in human [13]. The commonly cited radio-protective drug amifostine has multiple side effects including the hearing impairment [9, 14–16]. In this study, we investigated a new aminothiol radioprotector (PrC-210), which belongs to the family of aminothiol radioprotectors and is highly efficient and has fewer side effects, such as nausea and hypotension when administered orally or systemically [17, 18]. PrC-210 conferred 100% survival in rat and mouse models against a 100% lethal whole-body radiation dose (9.0 Gy) when administered 30–90 min before irradiation [17]. The aim of this study was to investigate whether PrC-210, administered prior to radiation, via intra-tympanic (IT) or intra-peritoneal (IP) routes, protects against radiation induced inner ear damage and hearing loss.

## Materials and Methods

All the experiments were conducted and approved by the Institutional Animal Care and Use Committee (IACUC protocol number 2D12106).

### Animals and Study Groups

Guinea pigs weighing 250–300 grams were obtained from Charles River (Wilmington, MA). All animals had ABRs performed at the beginning of the study. Animals with abnormal baseline ABR were excluded. Guinea pigs with normal hearing were divided into three groups (Table 1). Radiation doses were confirmed by InLight® nanoDot™dosimeters, designed for use in single point radiation with a dose operating range of 10 μGy to >100 Gy. Animals from each group had ABRs performed on day 6 and day 12. All animals were monitored daily for weight loss, signs of dehydration, abnormal behavior, aspect of the fur and routine health maintenance. Animals that were exhibiting signs of anorexia and dehydration before the end of the study were euthanized utilizing Fatal Plus (Vortech Pharmaceuticals, Dearborn, MI USA) via intracardiac injection, then decapitated.

**Table 1:** Distribution of Guinea pigs into various study groups.

	Radiation (cGy)			PrC-210		Number of animals	Death of animals		Number of Cochleae
	Left	Right	Cumulated	IT (185mg/kg)	IP (93.6mg/kg)		Week 1	Week 2	
Group 1	2000	2000	3000	No	No	8	0	1	14
Group 2	2000	2000	3000	Yes	No	5	0	1	8
Group 3	2000	2000	3000	No	Yes	6	0	2	8
Total						19	0	4	30

### Auditory Brainstem Response (ABR)

ABR testing was performed under general anesthesia. Animals were positioned in a faraday cage and needle electrodes were placed on the vertex and behind each pinna. Frequency specific tone pips at 8, 16 and 32 kHz were delivered to the ear in 10 dB increments using high frequency transducers (MO14600) inserted into the ear canals [19]. Waveforms were recorded using an Intelligent Hearing Systems Smart-EP (Miami FL, USA). Thresholds were defined as the lowest level at which a reproducible response could be obtained. ABRs were performed prior to intra-tympanic (IT) injection or intra-peritoneal (IP) injections (day 0). Then, ABR evaluations were performed on day 6 and 12.

### Drug Administration

Animals were initially anesthetized using inhalational of 2–3% Isoflurane /oxygen, then kept asleep through nose mask using 1–2% isoflurane/oxygen during the hearing test and for drug injection. A surgical otomicroscope and speculum were utilized to identify the tympanic membrane. For Group 2: using a 1ml syringe and a 27G 1.5inch sterile needle, 100 μl of the PrC-210 solution at 185mg/kg, was injected IT. For Group 3: PrC-210 was given IP (93.6 mg/kg). The total amount of Prc-210 injected through IT was more important the IP injection as we are we are usually loosing some solution when we are injecting it through the intra-tympanic membrane. Less than one hour after drug administration, the animals were taken to the cesium irradiator equipment [17].

### Radiation protocol

**Fig 1:**
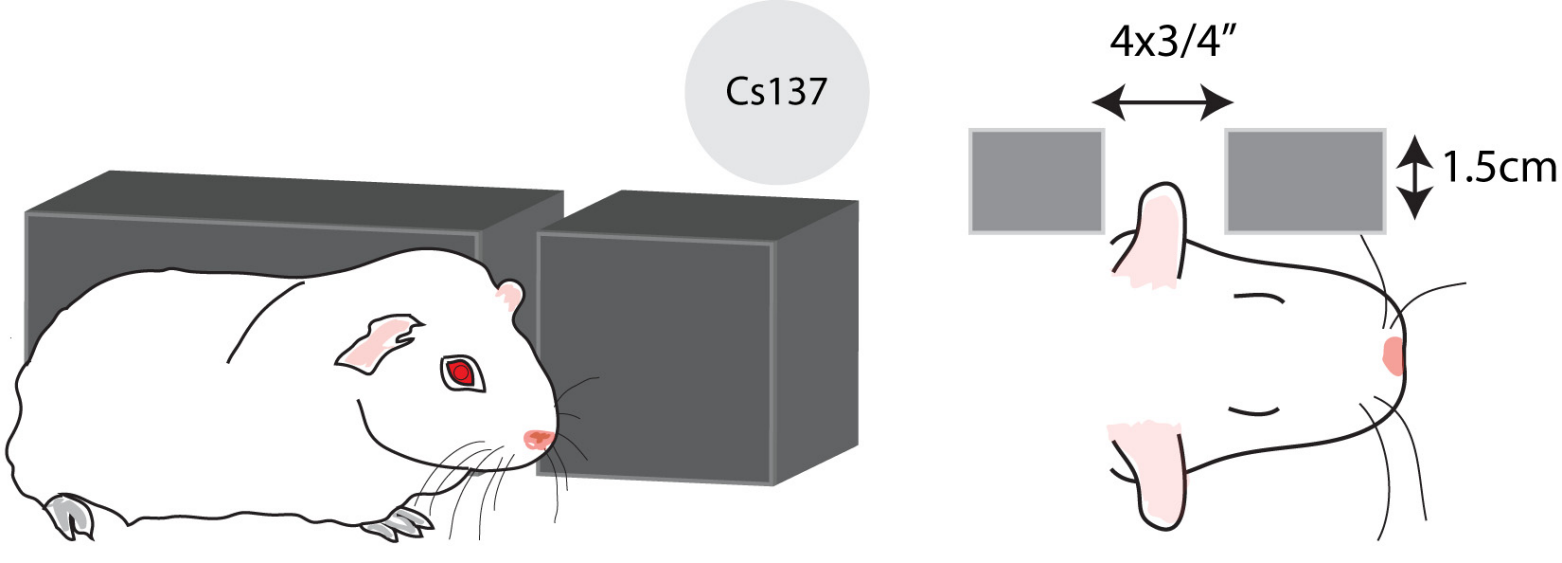
Radiation shielding devices. Diagram showing the lead shield that was used in order to protect the sedated guinea pigs. A 4x¾ “opening was drilled, so that the ear/auricle region of the guinea was exposed to radiation. Guinea pigs were irradiated with a Cs-137 irradiator. Sedated guinea pigs were placed on an elevated stage at the second position of the irradiator, behind the lead shield.

Prior to partial cranial irradiation, Ketamine (50mg/kg) and xylazine (6mg/kg) were administered subcutaneous to keep the animals immobilized in the prone position while in the cesium irradiator. A lead shield was constructed, 12” x 12” and 1.5 cm thick (Fig 1) to provide ~2 half value layers (HVL) of radiation shielding to the guinea pigs. A 4x¾ inch opening was drilled and tested by the physics team to confirm the output factor. Guinea pigs were irradiated with a Cs-137 irradiator (Mark I-68 A, J.L. Shepherd and Associated, San Fernando, CA). Sedated guinea pigs were placed on an elevated stage of the irradiator, behind the lead shield, exposing only the ear/auricle region (Fig 1). Each ear received 2000 cGy at a dose rate of 1.6 Gy/minute (total 12.5 minutes exposure, each side). The guinea pigs were turned to expose each side. During the radiation-planning phase of this trial, InLight® nanoDot™dosimeters were placed on the guinea pigs to confirm dosing. The results were consistent with measurements performed during planning. When measured with InLight® nanoDot™dosimeters, the intended planning dose was 1000 cGy for each cochlea: the left side entrance dose was 665 cGy and the exit dose was 424 cGy. The second trial yielded nanodot^TM^ results of 1111 cGy for a combined entrance and exit dose. The third trial yielded a combined entrance and exit dose of 1062 cGy.

A dose escalation trial was performed to assess the radiation dose needed for cochlear hair cell death at day 7. According to radiobiology literature, higher radiation doses achieve more significant radiation induced cochlear hair cell loss [9]. In guinea pigs, a single dose of 350 cGy is enough to promote hair cell death 30 days after irradiation [9]. A fractionated dose of 3300 cGy induces apoptosis of hair cells 72 hours after guinea pigs were irradiated but the percentage of hair cell death was not quantified [11]. In this dose escalation trial, we observed hair cell death 72 hours after guinea pigs received a cumulative dose of 1000 cGy (0.85% of dead hair cells) (S1 Fig). The hair cell death was increased when guinea pigs received a dose of 1000 cGy every 24h for 3 days but the number of dead hair cell was very low and cannot explain by itself the hearing loss induced by the radiation (2.5% of dead hair cells). In this study, due to animal irradiation schedule and logistics, we chose to give a single dose of 2000 cGy bilateraly resulting in a cumulative 3000 cGY dose for each temporal bone and evaluate hearing loss 6 and 12 days after irradiation.

### Postmortem analysis

In all groups, the surviving guinea pigs were euthanized utilizing Fatal Plus (Vortech Pharmaceuticals, Dearborn, MI USA) via intracardiac injection. Following euthanasia, temporal bones were harvested and fixed in 4% paraformaldehyde solution for 24 hours at 4°C. Excess bone was removed from around the cochlea. The cochlea was then decalcified using 0.25M EDTA (Promega, Madison, WI) at 4°C. After 48 hours, the cochlea was washed with 1X PBS and processed for paraffin embedding. The specimens were then embedded and sectioned at 6μm thickness, mounted and H&E stained.

### Antibodies

The following antibodies were used: rabbit anti-Myosin VII polyclonal antibody (1:200, Proteus Biosciences), Alexa Fluor-488 goat anti-rabbit (1:1000 dilution, Invitrogen).

### Immunostaining

The guinea pigs temporal bones were harvested and fixed in 4% PFA overnight at 4°C. After 3 washes with PBS, the temporal bones were incubated for 3 days in a PBS solution containing 0.25M EDTA. Cochleae were isolated and permeabilized with PBS containing 0.25% Triton X-100 for 1 hour. Cochleae were then blocked with 10% normal goat serum (NGS; Vector Labs) diluted in PBS for 1 hour. Cochleae were incubated overnight at 4°C with a primary antibody diluted in PBS containing 3% NGS. After washing cochleae were incubated with a secondary antibody for 1 hour in PBS solution containing 3% NGS. Rhodamine phalloidin was used at a 1:300 dilution for F-actin staining (Life Technologies, Carlsbad, CA). Nuclei were stained with DAPI (Molecular Probes). Cochleae were mounted using Prolong Gold antifade mounting medium (Molecular Probes). Organs of Corti were imaged using a confocal microscope (LSM 700, Carl Zeiss).

### Statistical analysis

Auditory Brainstem Response thresholds were analyzed using Prism 4 software (GraphPad software). Total average thresholds were analyzed using one-way analysis of variance (ANOVA). Average pure tone thresholds were compared using Student’s t-test. Hair cell loss data was analyzed using Student’s t-test. *, p< = 0.05; **, p< = 0.01; ***, p< = 0.001; n.s., non significant.

## Results

This study involved 19 total animals/ 38 total ears that were exposed to radiation. Four guinea pigs were euthanized as they showed signs of anorexia and dehydration, oral mucositis, stomatitis and moist confluent desquamation before the second week analyses. In the remaining 15 animals, there were 30 cochleae that were histologically examined.

### The aminothiol radioprotector PrC-210 did not cause inner ear cytotoxicity

To study the cytotoxicity of the aminothiol radioprotector PrC-210 on the inner ear, two guinea pigs received one intra-peritoneal (IP) or intra-tympanic (IT) injection of PrC-210. Guinea pigs were monitored daily for one week. The animals did not demonstrate vestibular dysfunction (head bobbing or head jerking movements). The guinea pig locomotion or coordination was not changed or altered. Seven days after injection of PrC-210, the animals were euthanized. Cochleae were dissected and immunostained with myosin VIIa, a sensory hair cell marker and phalloidin for the organ of Corti morphologic analysis. Guinea pigs treated with an IP or IT injections were noted to have normal cochleae and did not demonstrate quantitative hair cell loss in any of the cochlear turns (S2 Fig).

### Auditory radioprotective effects were demonstrated in guinea pigs that received pre-radiation Aminothiol PrC-210

**Fig 2:**
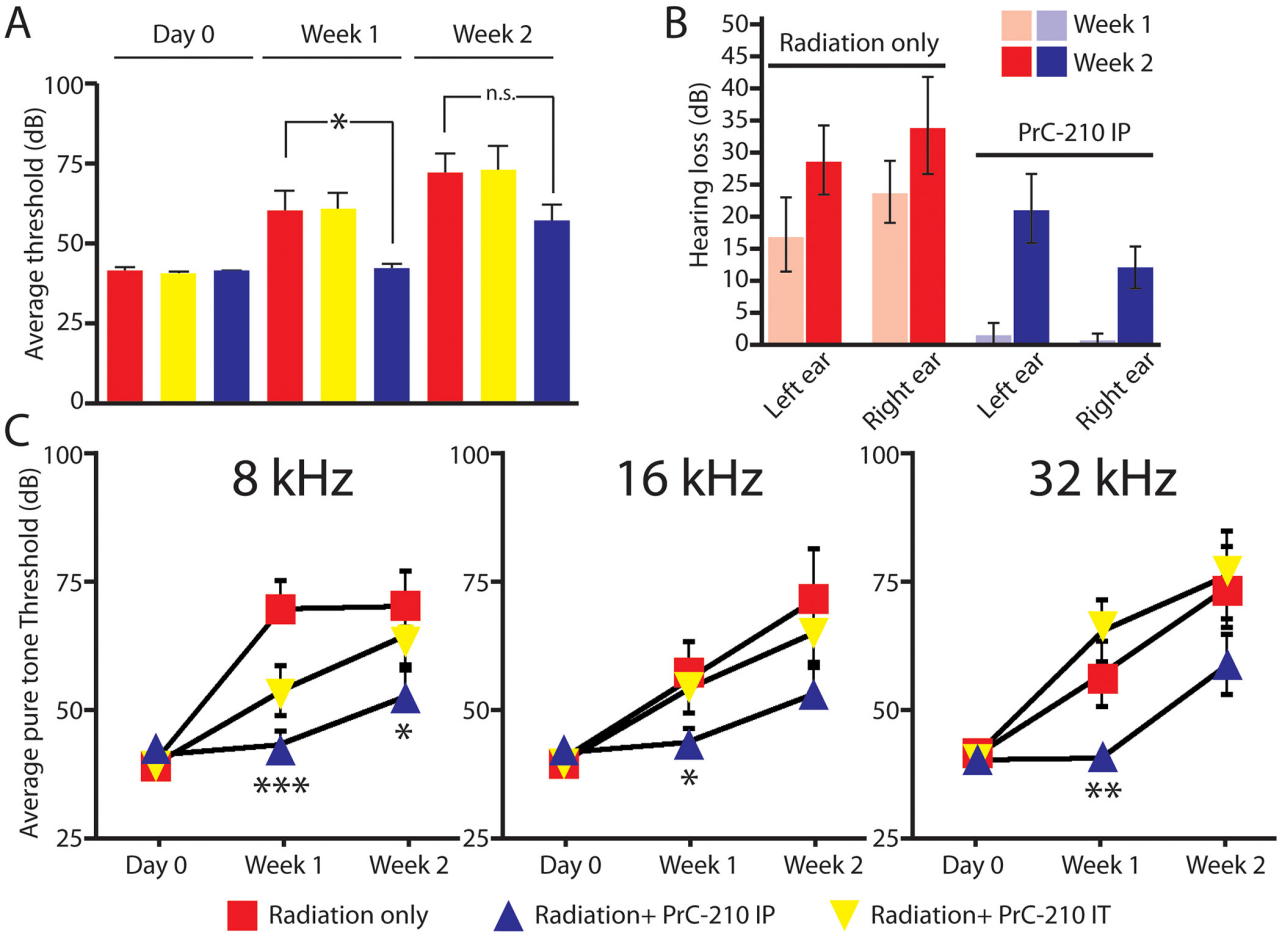
Auditory brainstem response (ABR) thresholds of irradiated adult guinea pigs treated by intra-peritoneal or intra-tympanic injection of PrC-210. (A) Total average thresholds at day 0, week 1 and week 2. Radiation: animals received 3000 cGy in each ear. Non-treated animals (red bars), animals treated with intra-peritoneal injection of PrC-210 (blue bars), animals treated with intra-tympanic injection of PrC-210 (yellow bars). (B) Hearing thresholds shift for the left and right ears of irradiated adult guinea pigs not treated with PrC-210 (Red) and treated by intra-peritoneal injection of PrC-210 (Blue). Total average hearing loss in dB at week 1 (light blue and light red bars) and week 2 (dark blue and dark red bars). (C) ABR pure tone thresholds of irradiated adult guinea pigs treated by an intra-peritoneal or intra-tympanic injection of PrC-210. Average pure tone thresholds at day 0, week 1 and week 2 measured at 8kHz, 16kHz and 32kHz. Radiation (Rad): animals received 3000 cGy in each ear. IP: intra-peritoneal injection of PrC-210 (blue triangles), IT: intra-tympanic injection of PrC-210 (inverted yellow triangles). Non-treated animals are indicated with red squares. Decibels (dB), *, p< = 0.05; **, p< = 0.01; ***, p< = 0.001, n.s., non significant.

The guinea pigs in this study were tested for hearing thresholds prior to PrC-210 and radiation exposure. To determine if PrC-210 has an auditory radioprotective effect, guinea pigs were divided into three groups. Group 2 received an intra-peritoneal (IP) dose. Group 3 received an intra-tympanic (IT) dose of PrC-210. Within one hour, the animals from all three groups were irradiated with a cumulative dose of 3000 cGy to each side. Every week for 2 weeks, ABRs click responses were recorded and average thresholds were compared to the non-treated arm (Group 1). Radiation induces an average of 20 dB hearing loss after 1 week in guinea pigs (Fig 2). This hearing loss was variable for the radiation treated arm but all the animals had a minimum of 5–10dB hearing threshold shift at week one. When animals received an intra-tympanic dose of PrC-210 just before irradiation (Fig 2; Rad+PrC-210 IT), they had a hearing loss statistically not different from the non-treated arm (Fig 2). Interestingly, the animals that received one injection of intra-peritoneal PrC-210 had significantly improved hearing thresholds, showing that the PrC-210 had protective effects on the radiation-induced hearing loss when injected in the systemic circulation (Fig 2; Rad+PrC-210 IP). However, after 2 weeks the hearing thresholds of PrC-210 treated animals were not significantly different from the non- PrC-210 treated arm, showing that the protective effect of PrC-210 is limited, not sustained. Although, the significance is lost at two weeks, there was still a trend toward improved thresholds with the radiation + intra-peritoneal PrC-210 arm (Fig 2). The hearing thresholds shift of both ears was comparable within each arm, showing the reproducibility of the data (Fig 2).

### The aminothiol PrC-210 has a better radioprotector effects on the apical coil of the cochlea

Next, average pure tone thresholds were measured at 8 kHz, 16 kHz and 32 kHz for each group of guinea pigs. After 1 week, 8 kHz, 16 kHz and 32 kHz tone thresholds of the Rad+PrC-210 IP arm were significantly lower than the thresholds of the non-treated and Rad+PrC-210 IT arms (S2 and S3 Figs). After 2 weeks, the 8 kHz tone threshold of the Rad+PrC-210 IP arm was significantly lower than the thresholds of the non-treated and Rad+PrC-210 IT arms, whereas the 16 kHz and 32 kHz tone thresholds were not significantly different (S2 and S3 Figs). These results suggest that the aminothiol PrC-210 had a better radioprotector effect on the apical coil than on the medial and basal coils of the cochlea.

### The aminothiol PrC-210 prevents the inflammation and the degeneration of the spiral ganglion induced by the radiation

**Fig 3:**
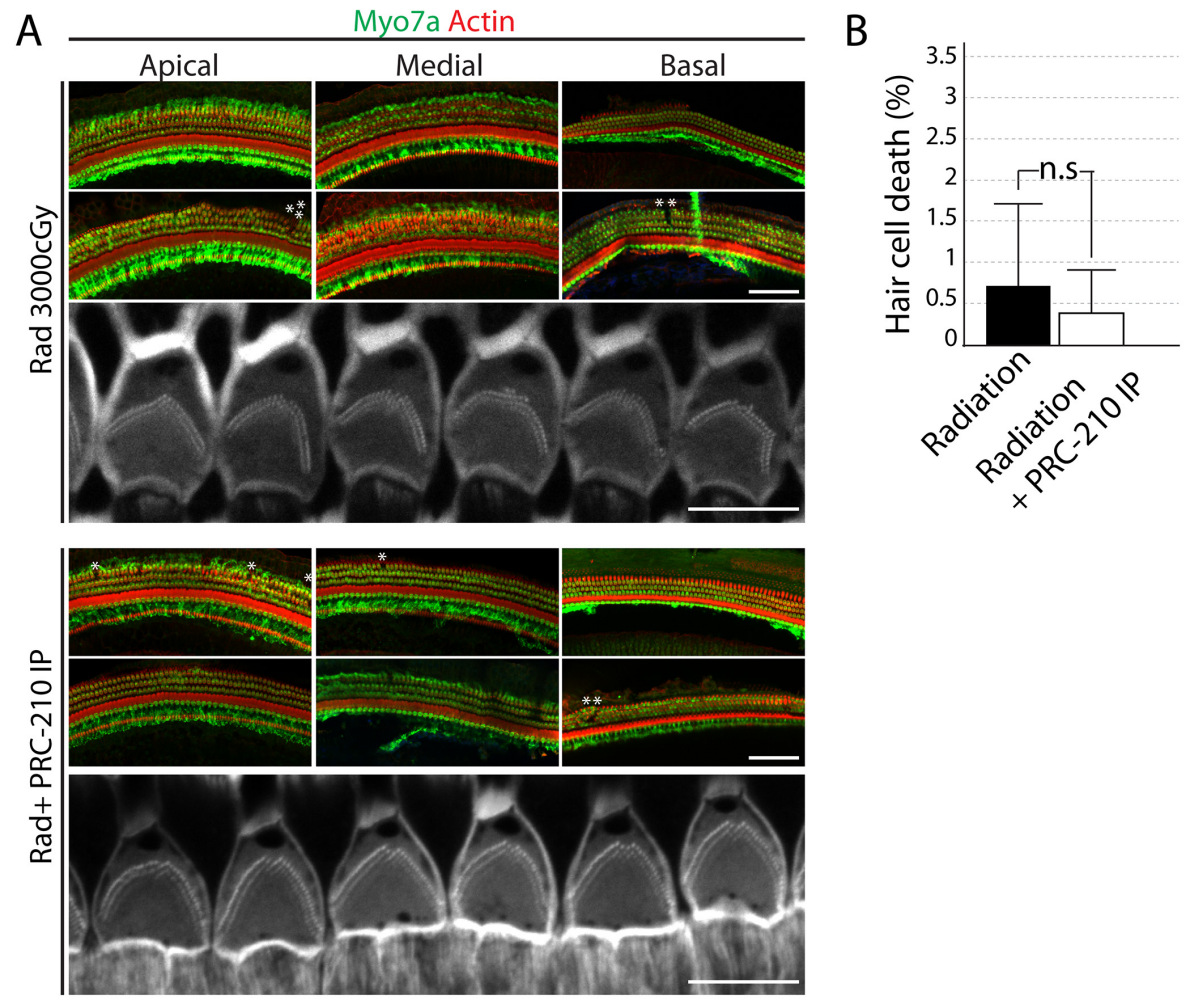
Cochlear sensory hair cell death in irradiated adult guinea pigs treated by an intra-peritoneal injection of PrC-210. (A) Cochleae from irradiated adult guinea pigs that were treated by an intra-peritoneal (IP) injection of PrC-210 were dissected at week 2. Cochleae were immunostained with Myosin VIIa antibody (Green), a hair cell marker and phalloidin (Red, Grey scale), an actin marker. Scale bar = 100 μm. Dead hair cells are labeled with a star (*) (B) The dead sensory hair cells were quantified and compared to the control group. n.s., non significant.

**Fig 4:**
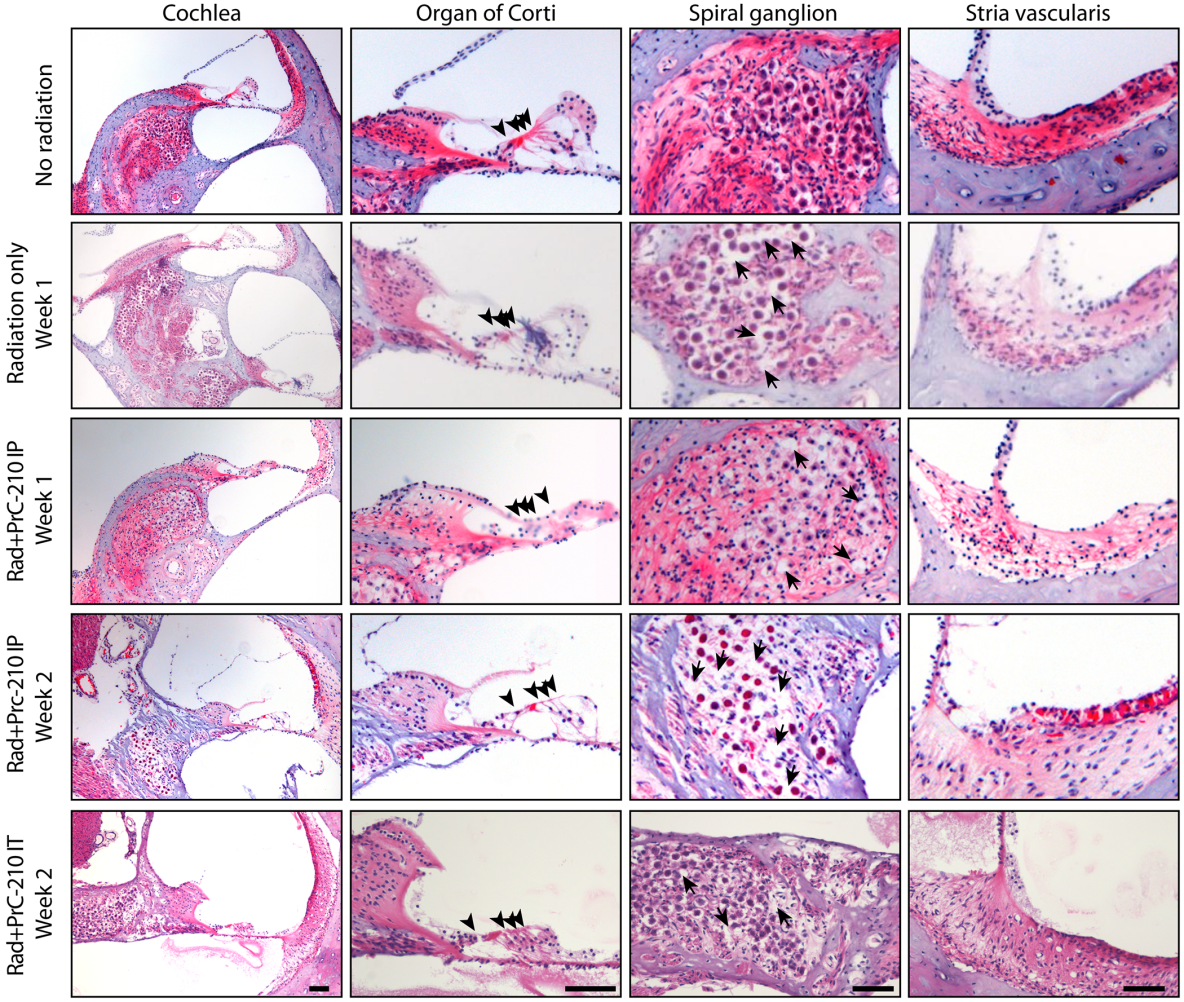
The aminothiol PrC-210 prevents the inflammation and the degeneration of the spiral ganglion induced by the radiation. Hematoxylin and Eosin staining of paraffin section of guinea pig cochleae treated by intra-peritoneal or intratympanic injection of PrC-210 before receiving a 3000 cGy radiation dose. Guinea pig cochleae from radiation only and PrC-210 IP groups were harvest one or two weeks after being irradiated and compared to a non-irradiated animal. Arrowheads indicate the sensory hair cells in the organ of Corti. Arrows indicate the inflammation spots in the degenerative spiral ganglion neurons. Scale bar: 100μm.

**Fig 5:**
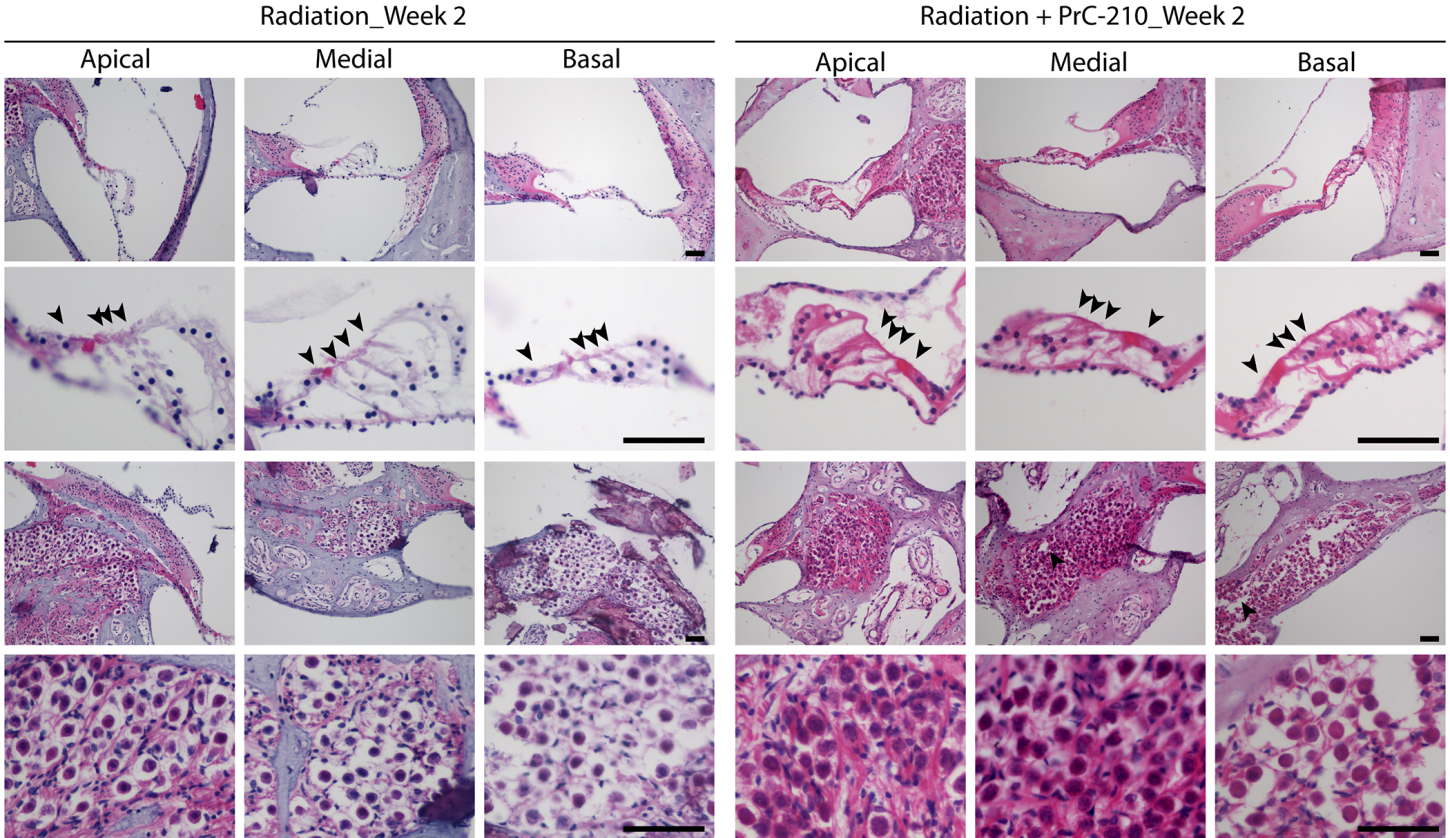
The aminothiol PrC-210 prevents the inflammation and the degeneration of the apical part of the spiral ganglion induced by the radiation. Hematoxylin and Eosin staining of paraffin section of guinea pig cochleae untreated and treated by intra-peritoneal injection of PrC-210 before receiving a 3000 cGy radiation dose. Guinea pig cochleae from radiation only and PrC-210 IP groups were harvest at two weeks after being irradiated. Arrowheads indicate the sensory hair cells in the organ of Corti. Scale bar: 100μm.

To identify the cause of the radiation-induced hearing loss, we analyzed the morphology of the organ of Corti and the spiral ganglion at 1 week and 2 weeks after the 3000 cGy dose of radiation. We did not observe any obvious deficit in the morphology of the organ of Corti after radiation exposure (Fig 3). No significant hair cell loss in any region of the cochlea coil was observed in any group (Figs 3 and 4). High magnification images are showing that the structure of the stereociliary bundle is not affected by the radiation (Figs 3 and 4). We also analyzed the morphology of cochlea, and the spiral ganglion of irradiated animals on hematoxylin/eosin stained paraffin sections as compared to non-irradiated animals (Fig 4). The spiral ganglion of irradiated/non-PRC-210 treated animals show signs of hydropic and vacuolar degeneration as compared to control group, which did not receive any radiation (Fig 4, arrows). When, animals were treated with intra-peritoneal injection of PrC-210, the spiral ganglion was preserved up to 1 week after the radiation. However, the spiral ganglion morphology revealed early signs of degeneration, including vacuoles in the cell body (Fig 4). Two weeks after the radiation, the spiral ganglion of PrC-210 treated animals presented a gradient of degeneration of the spiral ganglion along the apico-basal axis of the cochlea (Fig 4 and Fig 5).

## Discussion

In this pilot study, we demonstrate that intra-peritoneal and intra-tympanic injections of Prc-210 have no apparent cytotoxic effects on the inner ear. A single systemic injection of PrC-210 preserved hearing function of irradiated guinea pigs for one week. When injected into the systemic circulation, the aminothiol PrC-210 efficiently reduced the inflammation of the spiral ganglion and preserved the inner ear morphology. We hypothesize that the accessibility to the spiral ganglion by PrC-210 is better when injected into the systemic circulation, as the intra-tympanic injections of PrC-210 were not efficient. The investigators of this study presume that PrC-210 reaches the inner ear through the cochlear artery, reduces the oxidative stress in the spiral ganglion and or/ hair cells, and increases the survival of cells. The hearing preservation by PrC-210 was better in the low frequency than in the high frequencies. Basal hair cells, which are activated at high frequencies, are also more sensitive to chemicals and aging and potentially to the oxidative stress. The dose of PrC-210 might not be sufficient to prevent the death or the functionality of those cells. The radioprotector effect of PrC-210 was not sustained at 15 days after radiation, indicating that multiple injections of the drug might be suitable and might improve the radioprotection in the time.

## Supporting Information

S1 Fig Dose escalation trial. Guinea pigs were exposed to different doses of radiation (A, B, C and D) to assess the radiation dose needed for cochlear hair cell death at week 1 or week 2. Cochleae were immunostained with Myosin VIIa antibody (Green), a hair cell marker, phalloidin (Red), an actin marker and DAPI (Blue), a nucleus marker. Asterisks indicate the degenerated hair cells. Scale bar = 100 μm.
(TIF)

S2 Fig The aminothiol radioprotector PrC-210 is not toxic for the inner ear. Cochleae from adult guinea pigs that were treated by an intra-peritoneal (IP) or intra-tympanic (IT) injection of PrC-210 were dissected at week 1. Cochleae were immunostained with Myosin VIIa antibody (Green), a hair cell marker, phalloidin (Red), an actin marker and DAPI (Blue), a nucleus marker. Scale bar = 100 μm.
(TIF)

S3 Fig ABR pure tone traces of irradiated adult guinea pigs treated by an intra-peritoneal injection of PrC-210. ABR pure tone traces were measured at 8 kHz, 16 kHz and 32 kHz and at day 0, week 1 and week 2. Radiation: animals received a radiation dose of 3000 cGy in each pinna. IP: intra-peritoneal injection of PrC-210, The hearing thresholds are shown with red traces. Hearing thresholds are in Decibels (dB).
(TIF)
