# Supplementary figures and images for: Radioprotective Effect of Aminothiol PrC-210 on Irradiated Inner Ear of Guinea Pig
Source: PLoS One. 2015 Nov 23;10(11):e0143606. doi: 10.1371/journal.pone.0143606 (PMC4657906; doi:10.1371/journal.pone.0143606)

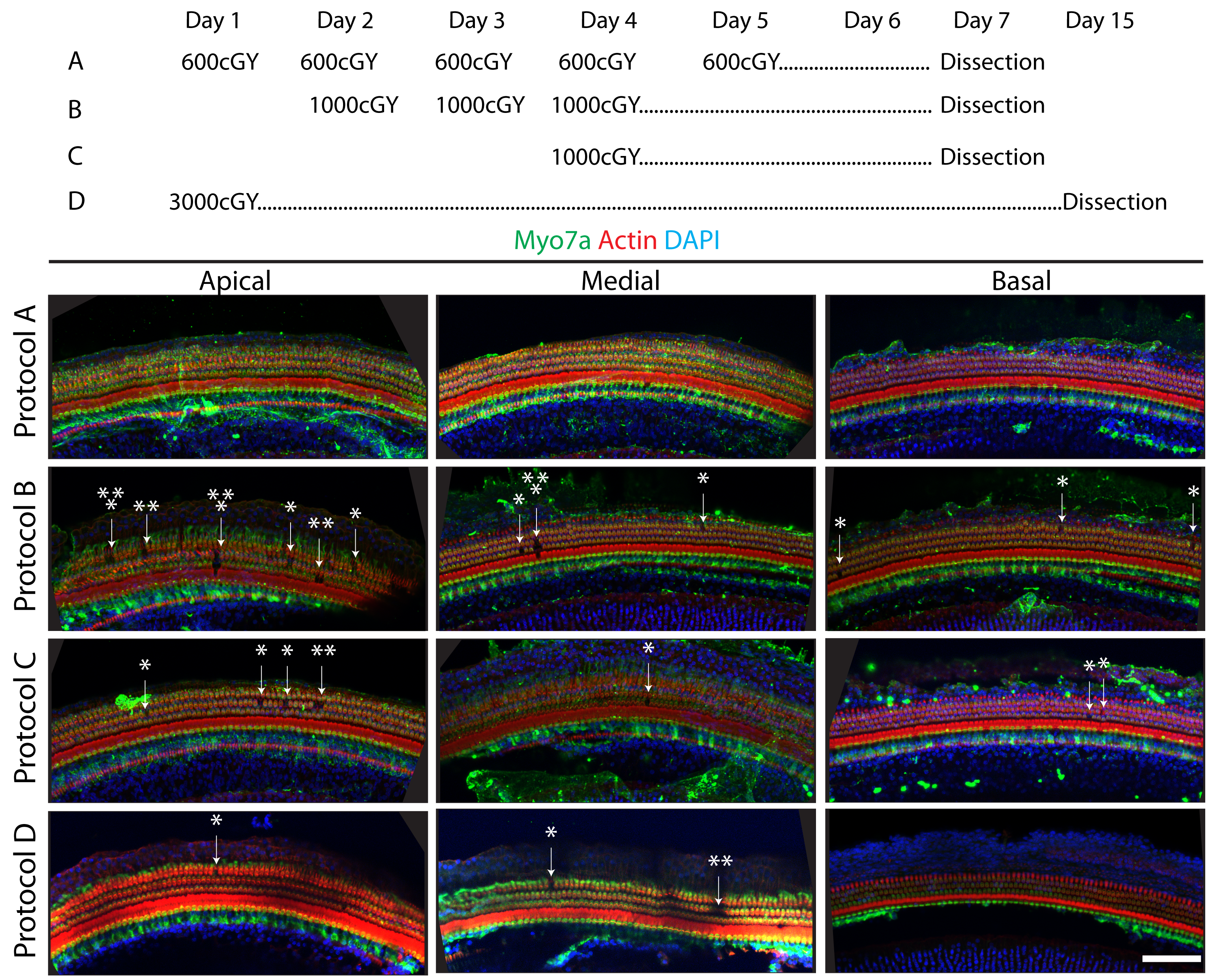

Supplement: S1 Fig — Guinea pigs were exposed to different doses of radiation (A, B, C and D) to assess the radiation dose needed for cochlear hair cell death at week 1 or week 2. Cochleae were immunostained with Myosin VIIa antibody (Green), a hair cell marker, phalloidin (Red), an actin marker and DAPI (Blue), a nucleus marker. Asterisks indicate the degenerated hair cells. Scale bar = 100 μm. (TIF) [file pone.0143606.s001.tif]

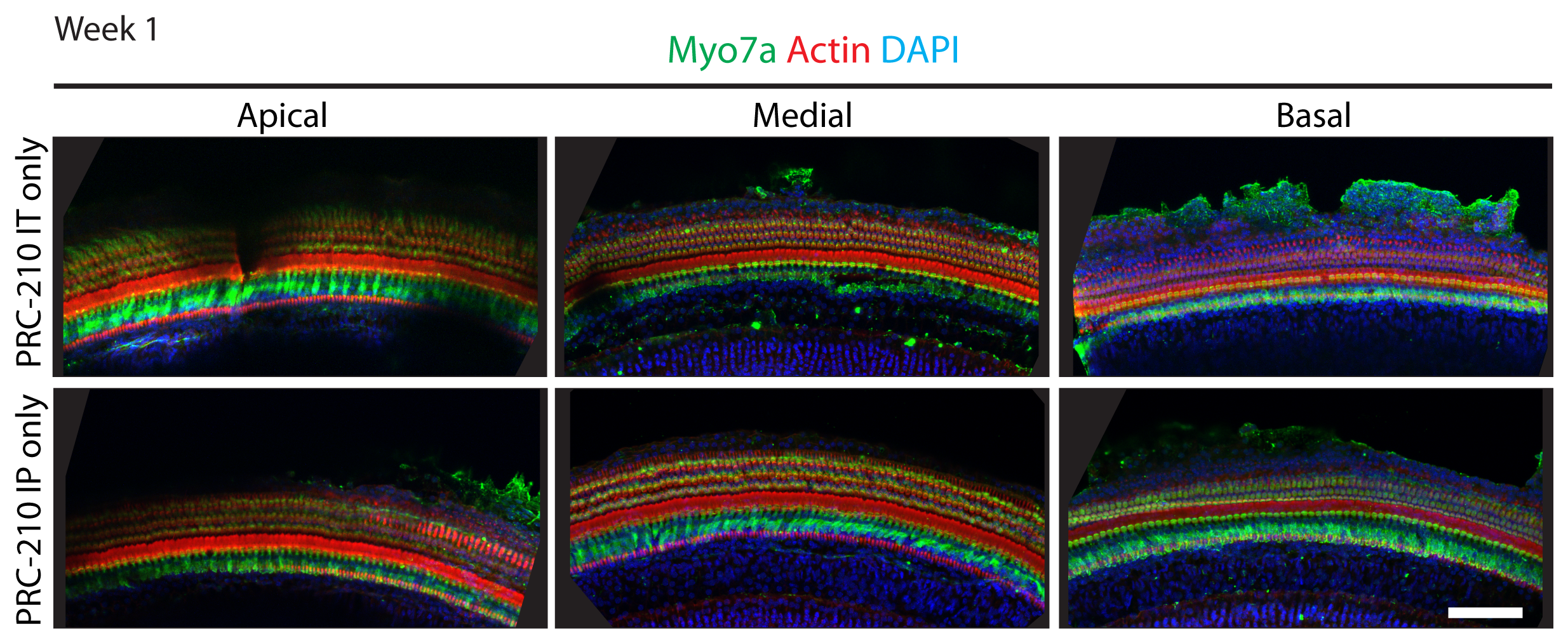

Supplement: S2 Fig — Cochleae from adult guinea pigs that were treated by an intra-peritoneal (IP) or intra-tympanic (IT) injection of PrC-210 were dissected at week 1. Cochleae were immunostained with Myosin VIIa antibody (Green), a hair cell marker, phalloidin (Red), an actin marker and DAPI (Blue), a nucleus marker. Scale bar = 100 μm. (TIF) [file pone.0143606.s002.tif]

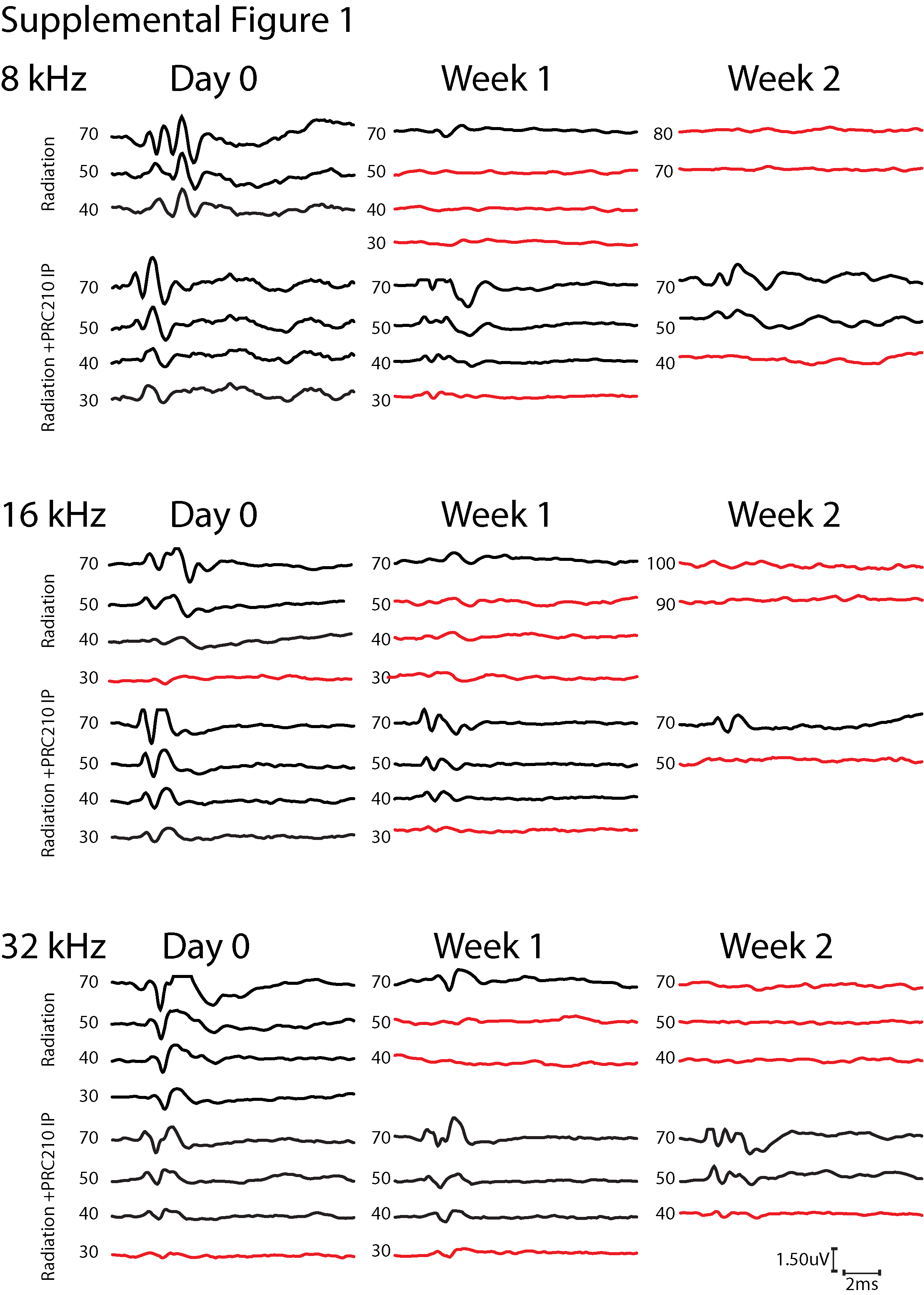

Supplement: S3 Fig — ABR pure tone traces were measured at 8 kHz, 16 kHz and 32 kHz and at day 0, week 1 and week 2. Radiation: animals received a radiation dose of 3000 cGy in each pinna. IP: intra-peritoneal injection of PrC-210, The hearing thresholds are shown with red traces. Hearing thresholds are in Decibels (dB). (TIF) [file pone.0143606.s003.tif]
